# Spectral changes in skin blood flow during pressure manipulations or sympathetic stimulation

**DOI:** 10.1113/EP091706

**Published:** 2024-04-20

**Authors:** Natalia S. Lima, Yi‐Ting Tzen, Philip S. Clifford

**Affiliations:** ^1^ Integrative Physiology Laboratory University of Illinois at Chicago Chicago Illinois USA; ^2^ University of Texas Southwestern Medical Center Dallas Texas USA

**Keywords:** myogenic, neurogenic, short‐time Fourier transformation, spectral analysis

## Abstract

Skin blood flow is commonly determined by laser Doppler flowmetry (LDF). It has been suggested that pathophysiological conditions can be assessed by analysis of specific frequency domains of the LDF signals. We tested whether physiological stimuli that activate myogenic and neurogenic mechanisms would affect relevant portions of the laser Doppler spectrum. LDF sensors were placed on the right forearm of 14 healthy volunteers for myogenic (six females) and 13 for neurogenic challenge (five females). Myogenic responses were tested by positioning the arm ∼50° above/below heart level. Neurogenic responses were tested by immersing the left hand into an ice slurry with and without topical application of local anaesthetic. Short‐time Fourier analyses were computed over the range of 0.06 to 0.15 Hz for myogenic and 0.02 to 0.06 Hz for neurogenic. No significant differences in spectral density were observed (*P* = 0.40) in the myogenic range with arm above (7 ± 54 × 10^−4 ^dB) and below heart (7 ± 14 × 10^−4 ^dB). Neurogenic spectral density showed no significant increase from baseline to cold pressor test (0.0017 ± 0.0013 and 0.0038 ± 0.0039 dB; *P* = 0.087, effect size 0.47). After application of anaesthetic, neurogenic spectral density was unchanged between the baseline and cold pressor test (0.0014 ± 0.0025 and 0.0006 ± 0.0005 dB; *P* = 0.173). These results suggest that changes in the myogenic and neurogenic spectral density of LDF signals did not fully reflect the skin vascular function activated by pressure manipulation and sympathetic stimulation. Therefore, LDF myogenic and neurogenic spectral density data should be interpreted with caution.

## Abstract (highlights)

- **What is the central question of this study?**
  Does spectral analysis of laser Doppler skin blood flow signals reflect physiological stimuli that activate myogenic and neurogenic vascular mechanisms?
- **What is the main finding and its importance?**
  Spectral densities of laser Doppler skin blood flow in the myogenic and neurogenic ranges do not fully reflect the skin vascular function activated by the physiological stimuli of pressure manipulation and sympathetic stimulation, respectively. Therefore, LDF myogenic and neurogenic spectral density data should be interpreted with caution.

## 1 INTRODUCTION

Skin blood flow is commonly used to investigate thermoregulation, cardiovascular integration, and as a tool to evaluate generalized microvascular function in healthy and clinical populations (Cui et al., 2010; Holowatz et al., 2008; Johnson et al., 2014). In fact, regulation of skin blood flow is a complex interaction among many physiological factors including local and autonomic responses (Cracowski & Roustit, 2020).

The myogenic response is the physiological characteristic of vascular smooth muscle cells to contract after an increase in transmural pressure (Bayliss, 1902; Johnson, 1991). The mechanism of this response has been well studied and includes the stepwise events of increased transmural pressure, stretch‐induced smooth muscle depolarization, depolarization and Ca^2+^ influx through L‐type Ca^2+^ channels, Ca^2+^ and calmodulin‐dependent phosphorylation of myosin light chain kinase, and actin/myosin‐based contraction (Carberry et al., 1992; Clifford, 2011; Hong & Lee, 2017; Pesic et al., 2004). Thus, the myogenic response to elevated intravascular pressure leads to vasoconstriction and a reduction in blood flow, and the myogenic response to lowered intravascular pressure leads to vasodilatation and an increase in blood flow.

In addition to local control, the skin is under the influence of autonomic control. Vasodilatation can occur through the activation of sympathetic cholinergic nerves whereas vasoconstrictor responses are mediated by sympathetic adrenergic nerves and the release of noradrenaline and neuropeptide Y (Crandall et al., 2002; Hodges et al., 2009; Stephens et al., 2004). These competing mechanisms play an important role in the regulation of arterial blood pressure.

Changes in skin blood flow can be measured non‐invasively using laser‐Doppler flowmetry (LDF) (Hurr et al., 2018; Kellogg et al., 1998; Kenney et al., 2014; Lima et al., 2022; Minson, 2010; Wolf et al., 2020). Spectral analysis of time–frequency domains is a method that decomposes the rhythmic activities of the skin blood flow responses and assigns them to potential corresponding vascular mechanisms (Bracic & Stefanovska, 1998; Stefanovska et al., 1999). Early work in this field suggested that different frequency intervals correspond to different vascular control mechanisms. These frequencies are represented as: 0.005–0.0095 Hz (endothelial), 0.0095–0.02 Hz (metabolic), 0.02–0.06 Hz (neurogenic), 0.06–0.15 Hz (myogenic), 0.15–0.4 Hz (respiratory) and 0.4–1.6 Hz (cardiac) (Bracic & Stefanovska, 1998; Kastrup et al., 1989; Kvandal et al., 2006; Kvernmo et al., 1998; Stefanovska et al., 1999). Spectral analysis of time–frequency domains of the laser Doppler signal has been used to study mechanisms of skin blood flow changes with different stimuli in healthy and clinical populations (Geyer et al., 2004; Jan et al., 2009; Tzen et al., 2018). However, there has been limited research verifying that physiological challenges result in changes in power within the relevant frequency range. To further corroborate the vascular mechanisms associated with two specific laser Doppler spectral frequencies, we tested the myogenic responses by manipulating vascular pressure and the neurogenic responses with a cold pressor test. We hypothesized that these stimuli would produce measurable changes within the relevant frequency ranges.

## 2 METHODS

### 2.1 Ethical approval

All study procedures were approved by the Institutional Review Board at the University of Illinois at Chicago (2019‐0299) and in accordance with guidelines set forth by the *Declaration of Helsinki* except for registration in a database. Verbal and signed written consents were obtained from each participant before participating in this study.

### 2.2 Participants

Healthy participants (22–38 years) were recruited to participate in this study. Participants were excluded if they reported smoking, presence of cardiovascular, pulmonary, metabolic and neurological diseases. Blood pressure cutoff for hypertension was >140 (systolic)/90 (diastolic) mmHg and hypotension was <90 (systolic)/60 (diastolic) mmHg. Other exclusion criteria obesity (body mass index >35 kg/m^2^), anti‐inflammatory medication and pregnancy. We asked participants to refrain from exercise, food, beverages and caffeine for at least 8 h before their test. Female participants were tested within 1–7 days after menses began or during the placebo phase if taking oral contraceptives to control for hormonal variation. We assessed height and weight using a standard stadiometer and an electronic scale, respectively.

### 2.3 Experimental procedures

Participants lay supine in a quiet and temperature‐controlled room for 10 min before instrumentation. A finger cuff photoplethysmography sensor (Finometer Pro, Finapres Medical System, Amsterdam, Netherlands) was placed on the middle finger of the right hand for continuous beat‐by‐beat arterial pressure measurement. Two Peltier‐controlled thermomodules (1.5 cm diameter) and two laser Dopper flow (LDF) probes (Moor Instruments Inc., Wilmington, DE, USA) were placed 10 cm above the wrist on the volar surface of the right forearm for continuous measurement of temperature and LDF. The temperature of the probes was maintained at 33°C throughout the entire protocol (Holowatz & Kenney, 2011; Lima et al., 2022). Parts A and B of the experimental procedures were conducted on different days with different participants.

### 2.4 Part A. Myogenic

After rest and instrumentation, myogenic reactivity in the human cutaneous microvasculature was tested by manipulating the local intravascular pressure in the microcirculation under the LDF sensors. This was accomplished by lowering the arm (∼50° below the heart) for 5 min then raising the arm (∼50° above the heart) for 5 min in a counterbalanced order (Jasperse et al., 2015). The subject's arm was supported by a stationary device (JawStand XP, Rockwell, Charlotte, NC, USA), and tilted to the appropriate angles. Local pressure in the forearm at the two positions was estimated by adjusting for differences in the hydrostatic column between the heart and mid‐forearm laser Doppler sensors. Arterial pressure and LDF were measured continuously in 14 participants.

### 2.5 Part B. Neurogenic

Sympathetic responsiveness in human cutaneous microvasculature was evaluated in 13 participants by immersing the left hand up to the wrist in an ice water slurry (cold pressor test) while the right arm was maintained at heart level. To study whether alterations in cutaneous microvascular reactivity in response to a sympathetic stimulus result from inherent properties of vascular smooth muscle cells or cutaneous neural reflex, the protocol was repeated after the application of anaesthetic cream (EMLA, 2.5% lidocaine and 2.5% prilocaine) on the skin to block cutaneous vasoconstrictor nerves.

After instrumentation, subjects rested quietly before collecting 1 min baseline LDF and arterial pressure. Then, the cold pressor test was administered for 5 min. Afterwards, the hand was dried and placed on a warming pad until the subject was comfortable. The positions of the laser Doppler flow probes were marked with a pen, and they were temporarily removed before the application of the anaesthetic cream. Five grams of anaesthetic cream was applied to the skin and secured with a transparent tape (Tegaderm™ Film, 10cm × 12cm) for at least 120 min. Loss of cutaneous sensation was verified by the response to a tactile stimulus (touch with a sharp needle) and the cream was wiped off. The laser Doppler flow probes were returned to their original positions and measurements were repeated.

### 2.6 Data analyses

All analog signals were continuously recorded in real time and stored at 1000 Hz using PowerLab (ADInstruments, Colorado Springs, CO, USA). Blood flows from the two LDF probes were averaged, and cutaneous vascular conductance (CVC) was calculated as LDF divided by the mean arterial pressure (MAP). Likewise, the short‐term Fourier analysis was performed for each probe separately (see below) and then averaged for each individual subject.

MATLAB (The MathWorks, Natick, MA, USA) was used for LDF signal processing. To ensure sufficient frequency resolution, all LDF signals were first down sampled to 0.5 Hz using code ‘decimate’. The LDF signals were then proceeded with short‐time Fourier analyses, using *S*_t_(ω) as the Fourier transform of signal *s*(τ) and *h*(τ − *t*) as the window function:

(1) $$P_{\mathrm{SP}}\left(t,\omega \right)\;={\left|S_{t}\left(\omega \right)\right|}^{2}\;={\left|\frac{1}{\sqrt{2\pi }}\int e^{-i\omega t}s\left(\tau \right)h\left(\tau -t\right)d\tau \right|}^{2}\;$$

Window length of 128 was selected. To calculate myogenic and neurogenic spectral densities, spectral densities between the frequency range 0.06–0.15 Hz and 0.02–0.06 Hz were integrated. For the purpose of statistical analyses, myogenic spectral density during the following time periods was integrated for arm below (420–540 s) and above (720–840 s) heart level. These two time periods were selected because the LDF signal was not affected by any transition of the arm position change. For the neurogenic spectral density calculation, spectral density during the following time periods was integrated for baseline (90–210 s), and cold pressor test (300–420 s). The time period selected for baseline LDF signal was to avoid any effect caused by the cold pressor test. The time period selected for the cold pressor test was to capture the first 2 min of the response in the cold pressor test. Equation (2) demonstrates the calculation of baseline neurogenic spectra density for the cold pressor test:

(2) $$P_{\mathrm{sp}-\omega }=\int_{\omega =0.02\;\mathrm{Hz}}^{0.06\;\mathrm{Hz}}\int_{T=300\;s}^{420\;s}\left(T,\omega \right)dTd\omega \;$$

### 2.7 Statistical analysis

All data are presented as means or medians ± SD. Normality was analysed using Kolmogorov–Smirnov test and logarithmically transformed when needed. A non‐parametric related‐samples Wilcoxon test was performed for statistical analysis of myogenic spectral range in Part A. Student's paired *t*‐test was used to test differences between arm up and arm down for LDF, local CVC and local MAP.

Related‐samples Friedman's two‐way analysis of variance test with a Wilcoxon post‐hoc test was used to compare the spectral density changes of skin blood flow for the cold pressor experiment. A two‐way ANOVA was used to test the differences in LDF, CVC and MAP for the cold pressor test.

Data analyses were carried out using SPSS Statistics version 24 (IBM Corp., Armonk, NY, USA) and an a priori significance was set at α < 0.05.

## 3 RESULTS

### 3.1 Part A. Myogenic

Participant characteristics for parts A and B are presented in Table 1. Local arterial pressure under the sensor was significantly different between arm above and below heart level (*P* < 0.001) (Table 2). As expected, skin blood flow and CVC were significantly reduced when the arm was positioned below the heart level (*P* = 0.001).

**TABLE 1:** Participant characteristics.

Characteristics	Group	Females	Males
Part A. Myogenic	*n* = 14	*n* = 6	*n* = 8
Age (years)	30 ± 6	28 ± 5	32 ± 6
Height (cm)	173 ± 8	166 ± 4	178 ± 5
Weight (kg)	78 ± 15	67 ± 11	86 ± 13
Body mass index (kg/m^2^)	26 ± 4	21 ± 3	27 ± 3
Part B. Neurogenic	*n* = 13	*n* = 5	*n* = 8
Age (years)	28 ± 6	26 ± 6	32 ± 6
Height (cm)	173 ± 8	169 ± 5	175 ± 8
Weight (kg)	75 ± 18	67 ± 11	82 ± 18
Body mass index (kg/m^2^)	25 ± 4	21 ± 3	26 ± 4

*Note*: Values are means ± SD; *n* = number of participants.

**TABLE 2:** Local haemodynamic responses with arm above and below heart level.

*n* = 14	Arm up	Arm down
LDF (au)	18.48 ± 9.48	13.43 ± 8.13^*^
Local CVC (au/mmHg)	0.28 ± 0.20	0.17 ± 0.11^*^
Local AP (mmHg)	80 ± 8	102 ± 8^*^

*Note*: Values are means ± SD. *P < 0.05 compared to Arm Up condition. Abbreviations: AP, arterial pressure; CVC, cutaneous vascular conductance; LDF, laser Doppler flow.

**FIGURE 1:**
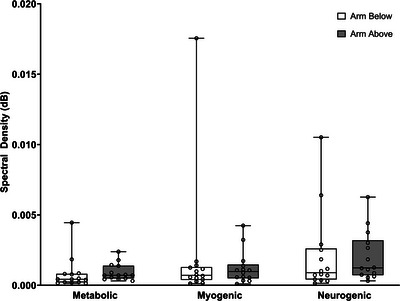
Metabolic, myogenic and neurogenic spectral densities with arm positioned above and below heart level. No significant differences in the myogenic spectral density were observed after increasing local pressure (arm below) or reducing local pressure (arm above); *n* = 14.

Figure 1 shows the effect of limb position on the metabolic, myogenic and neurogenic spectral densities. Statistical analysis did not detect significant changes in the myogenic spectral density between arm above and below heart level (*P* = 0.40).

### 3.2 Part B. Neurogenic

Under control conditions, cold pressor decreased CVC (*P* = 0.038) with no change in LDF (*P* = 0.833; Table 3). After the application of EMLA cream, there was no longer a decrease in CVC (*P *= 0.251) and LDF was significantly increased (*P* < 0.001). Arterial pressure increased during cold pressor in both the control and EMLA conditions (*P* < 0.001).

**TABLE 3:** Haemodynamic responses with and without application of anaesthetic cream (EMLA).

	Control		Topical anaesthetic	
Measure	Baseline	Cold pressor test	Baseline	Cold pressor test
LDF (au)	9.72 ± 2.97	9.71 ± 3.41	11.71 ± 4.98	14.98 ± 6.58^*#^
CVC (au/mmHg)	0.12 ± 0.04	0.10 ± 0.03^*^	0.13 ± 0.06	0.14 ± 0.07
AP (mmHg)	88 ± 11	97 ± 10^*^	93 ± 9	107 ± 10^*#^

*Note*: Values are means ± SD. *P < 0.05 versus baseline. ^#^ *P* < 0.05 versus control. Abbreviations: AP, arterial pressure; CVC, cutaneous vascular conductance; LDF, laser Doppler flow.

**FIGURE 2:**
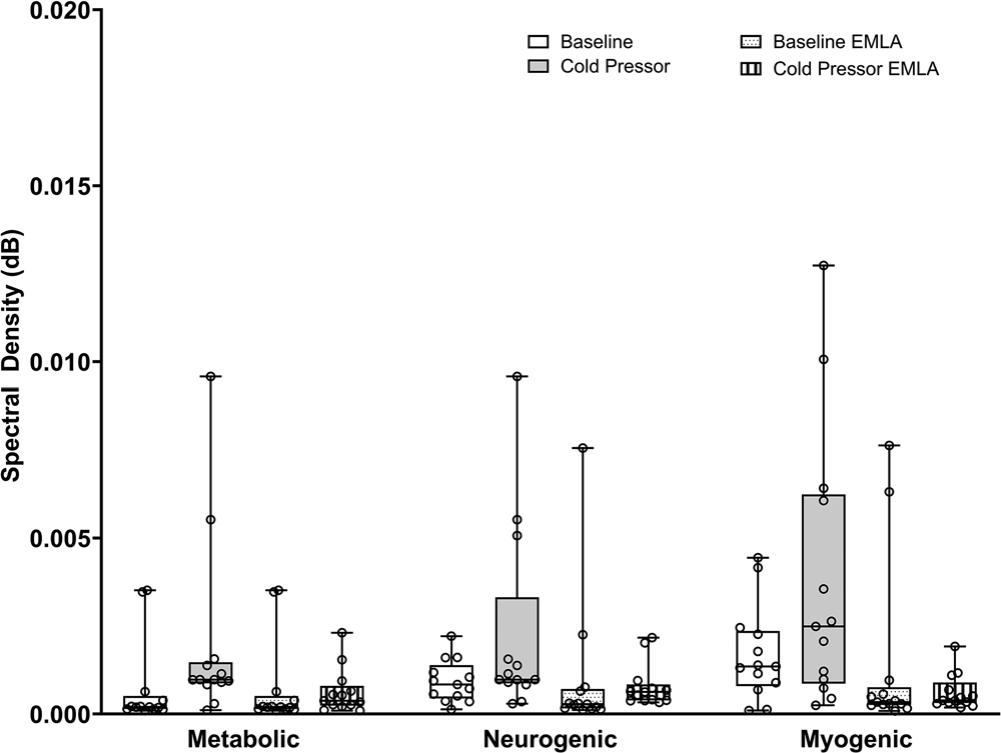
Metabolic, myogenic and neurogenic spectral densities at baseline and during a cold pressor test with and without application of anaesthetic cream. No significant differences were observed in the neurogenic spectral density during the cold pressor test before or after the application of the anaesthetic cream; *n* = 13.

The effect of cold pressor test on the metabolic, myogenic and neurogenic spectral densities before and after application of anaesthetic cream is shown in Figure 2. Neurogenic spectral density showed a medium effect size (0.47) but no statistical significance (*P* = 0.087) from baseline to cold pressor under the control condition. Individual data revealed that neurogenic spectral density increased in seven participants during the cold pressor test while no change (*n* = 4) or a decrease (*n* = 2) was observed in the rest of the group. After the application of EMLA cream, neurogenic spectral density remained similar between baseline and cold pressor test (*P* = 0.173).

## 4 DISCUSSION

The purpose of this study was to investigate whether challenging the myogenic and neurogenic responses of skin vasculature would be reflected in changes in the myogenic and neurogenic spectral densities. We challenged the myogenic response of skin vascular arteries by changing local intravascular pressure by raising and lowering the arm above and below the heart level. The results demonstrated no significant changes in the myogenic spectral density related to changes in intravascular pressure. The neurogenic response of skin microvessels was examined using a cold pressor test with and without topical application of a local anaesthetic cream to block nerve fibres in the skin. The results showed a medium effect size but no significant increase in the neurogenic spectral density during the cold pressor test. These findings suggest that LDF myogenic and neurogenic spectral density data should be interpreted with caution.

Based on earlier work on heart rate variability and local minima of the wavelet transform of the laser Doppler signal, five characteristic frequencies were identified. The frequency range 0.005–0.0095 Hz is associated with endothelial function, 0.0095–0.02 Hz with metabolic function, 0.02–0.06 Hz with neurogenic activity, 0.06–0.15 Hz myogenic response, 0.15–0.4 Hz respiration and 0.4–1.6 Hz cardiac function (Stefanovska et al., 1999).

To our knowledge, this is the first study attempting to corroborate that oscillations in the LDF signal between 0.06 and 0.15 Hz represent myogenic influences. Pressure manipulation was accomplished by raising and lowering the arm above and below the heart level. Raising the arm above the heart level reduces local pressure and lowering the arm increases local pressure. The myogenic response describes the capacity of vascular smooth muscle cells to constrict after an increase in transmural pressure or to dilate to a decrease in transmural pressure (Davis & Hill, 1999; Kuo et al., 1990; Roman & Dokkum, 2014). The vasoconstrictor response includes the stepwise events of increased transmural pressure, stretch‐induced smooth muscle depolarization, depolarization and Ca^2+^ influx through L‐type Ca^2+^ channels, Ca^2+^ and calmodulin‐dependent phosphorylation of myosin light chain kinase, and actin/myosin‐based contraction (Clifford, 2011; Hong & Lee, 2017; Johnson, 1991; Roman & Dokkum, 2014). The mechanism of the vasodilator response is less well understood (Davis et al., 2008). Our data showed no significant differences in the myogenic frequency range after an increase in local pressure, suggesting that spectral analysis of LDF cannot fully reflect the myogenic effects. Furthermore, previous research has shown that myogenic tone is absent in the skin blood vessels of healthy individuals (Crandall et al., 2002; Vissing et al., 1997). This stimulus had the expected haemodynamic effects of vasoconstriction confirming that there was vasoconstriction (decreased conductance) after increasing local pressure by lowering the arm below heart level. However, reductions of skin blood flow in response to position‐related changes in pressure have been attributed to the ‘venoarteriolar reflex’, rather than a local myogenic response (Crandall et al., 2002; Henriksen, 1977; Vissing et al., 1997). Based on the current data and the absence of myogenic responses in the skin microcirculation, it appears that myogenic changes in the skin vasculature cannot be inferred from spectral analysis of laser Doppler signals.

There is previous research regarding neurogenic oscillations in the LDF signal. Kastrup et al. (1989) identified oscillations in the LDF signal of resting subjects that they described as α‐ and β‐oscillations. The β‐oscillations were ascribed to neurogenic influences since they disappeared with local anaesthetic infiltration or ganglionic block. These β‐oscillations were observed at 0.025 Hz, which is within the neurogenic range. Our study is the first attempt to quantify neurogenic spectral density under conditions with a potent neurogenic stimulus. Neurogenic activity was amplified with a cold pressor test since it is known that immersion of the hand in an ice slurry increases skin sympathetic activity in the median nerve of the contralateral arm (Fagius & Blumberg, 1985; Fagius et al., 1989). This stimulus had the expected haemodynamic effects of vasoconstriction (decreased CVC) and increased pressure. Nevertheless, the effects on neurogenic spectral density were variable (seven increased, two decreased, four no change). There was a trend for an increase in power with a medium effect size, but it was not statistically significant. Application of anaesthetic cream abolished reflex vasoconstriction and the increase in neurogenic spectral density. These results suggest that neurogenic spectral density does not fully reflect changes in neurogenic function.

Previous validation studies of LDF spectral analysis have focused primarily on the endothelial frequencies (0.005–0.0095 Hz). In young healthy subjects, iontophoresis of acetylcholine (endothelium dependent vasodilator) increased the relative amplitude in the endothelial frequency range more than sodium nitroprusside (endothelium independent vasodilator) (Kvernmo et al., 1999; Stefanovska et al., 1999). This finding was replicated by Kvandal et al. (2006) and extended by showing that arterial infusion of the NO blocker *N* ^G^‐monomethyl l‐arginine abolished the difference between the two dilators (Kvandal et al., 2006). These studies provide convincing evidence regarding the involvement of the endothelium of the skin microvasculature in oscillations at these frequencies.

Spectral analysis of the LDF signal has been used to characterize diverse clinical conditions such as aging, diabetes, wound healing, hypertension, and exercise training. Jan et al. (2009) identified an impaired vasodilatory response of skin microvessels in older adults and a significantly impaired neurogenic control mechanism with ageing. Another study from the same group showed that diabetic individuals have an impaired metabolic, neurogenic and myogenic response to a thermal challenge, which may increase the risk of foot ulcers (Jan et al., 2013). The contribution of skin blood flow to wound healing was studied with spectral analysis by Tzen et al. (2018). The results showed that low‐intensity vibration increased skin blood flow to the affected area and that myogenic mechanism may be associated with improvements in wound healing. No differences in spectral density across the different frequencies were found between controls and patients with newly diagnosed or established hypertension (Rossi et al., 2006). The influence of exercise training was studied in a cross‐sectional study comparing normally active control subjects with competitive long‐distance runners. Amplitudes in the myogenic and neurogenic frequencies were greater in the athletes (Bracic & Stefanovska, 1998). The above studies show the potential clinical utility of spectral analysis of skin blood flow, but the data from the current study suggests caution in interpreting the results of LDF spectral analysis in the myogenic and neurogenic range.

We must consider factors that could have influenced the results of our study. It is well known that there is considerable spatial variability in skin blood flow. We applied two LDF sensors to the skin of each participant and averaged the values within that subject to reduce data variability. After the first cold pressor test, the probe positions were marked with a permanent marker so they could be carefully applied on the same sites after treatment with the anaesthetic cream. In addition, statistical comparisons were paired such that each subject was compared with their data. Topical application of local anaesthetic was used to interrupt sympathetic impulses. Although we tested sensory blockade, it is known that local anaesthetics block smaller diameter fibres first, followed by sensory fibres, and then motor fibres (Gasser & Erlanger, 1929; Macfarlane et al., 2020). It was assumed that when sensory fibres were blocked the sympathetic arc of the local venoarteriolar reflex was also blocked.

### 4.1 Conclusions

This study demonstrates that short‐time Fourier transformation of the LDF signal does not reflect changes in the myogenic range with local pressure manipulation by changes in arm position. In addition, short‐time Fourier transformation of LDF signals does not fully reflect changes in the neurogenic range after a cold pressor stimulus. Therefore, the results of LDF spectral analysis for the myogenic and neurogenic control mechanisms in healthy and clinical populations should be interpreted with caution.

## AUTHOR CONTRIBUTIONS

Natalia S. Lima conceived and designed research, performed experiments, analysed data, interpreted results of experiments, prepared figures, drafted manuscript, and edited and revised manuscript. Yi‐Ting Tzen conceived and designed research, analysed data, interpreted results of experiments, and edited and revised manuscript. Philip S. Clifford conceived and designed research, interpreted results of experiments, prepared figures, drafted the manuscript, and edited and revised the manuscript. All authors have read and approved the final version of this manuscript and agree to be accountable for all aspects of the work in ensuring that questions related to the accuracy or integrity of any part of the work are appropriately investigated and resolved. All persons designated as authors qualify for authorship, and all those who qualify for authorship are listed.

## CONFLICT OF INTEREST

The authors have no conflicts of interest to disclose.

## FUNDING INFORMATION

None.

## Data Availability

Data supporting the results of the present study are available from the corresponding author upon reasonable request.
